# The relationship between self-cohesion and smartphone addiction: the mediating role of rejection sensitivity

**DOI:** 10.3389/fpubh.2023.1166864

**Published:** 2023-06-28

**Authors:** Mogeda El Sayed El Keshky, Huda Aseem, Amira Alzain, Yasser Abdelazim Abdelmawgoud Samak

**Affiliations:** ^1^Department of Psychology, Faculty of Arts and Humanities, King Abdulaziz University, Jeddah, Saudi Arabia; ^2^Department of Geography and GIS, Faculty of Arts, Assiut University, Assiut, Egypt

**Keywords:** smartphone addiction, self-cohesion, rejection sensitivity, mediation analysis, Saudi Arabia

## Abstract

**Background:**

The increasing use of smartphones with attractive applications has yielded concerns over problematic overuse, also called smartphone addiction, thus creating a need to investigate the antecedents and pathways of this addiction.

**Objective:**

The aim of this study was to investigate the relationships between the self-cohesion dimensions of presence and consistency and smartphone addiction, and the potentially mediating role of rejection sensitivity.

**Methods:**

A total sample of 910 respondents (58% females, mean age = 34.9, SD = 13.6) participated in the study. They completed a questionnaire including demographic characteristics and queries about frequency and duration of smartphone use, as well as the Self-Cohesion Scale, the Short Version of Smartphone Addiction Scale, and the Rejection Sensitivity RS-Adult Questionnaire. Structural equation modeling was used to examine the relationships in question.

**Results:**

The findings indicated that smartphone addiction was positively correlated with rejection sensitivity and negatively correlated with the self-cohesion dimensions of presence and consistency. Further, both presence and consistency were negatively associated with smartphone addiction and rejection sensitivity negatively mediated these relationships.

**Conclusion:**

Self-cohesion and rejection sensitivity appear to be important predictors of smartphone addiction. Efforts to counteract smartphone addiction should, therefore, endeavor to increase smartphone users’ self-cohesion and reduce their rejection sensitivity.

## 1. Introduction

Smartphones have become an important part of our daily lives and the number of smartphone users continues to increase. The number of smartphone users worldwide in 2022 approached double that of 2016, growing from 3.7 billion to 6.6 billion (1). In Saudi Arabia in particular, there were 23.4 million smartphone users, or 71% of the population, in 2017, 33.1 million users, or 92% of the population, in 2022, and the number of users is expected to grow to 36.2 million in 2025 (2).

Smartphones are equipped with haptic screens, access to the internet via Wi-Fi or cellular networks, an enormous variety of applications, cameras, GPS, and more, which makes them suitable for multiple purposes, including not only communication but also entertainment and education (3). Further, it has been postulated that specific smartphone applications can offer effective support for certain health issues, such as alcoholism recovery (4) and diabetes self-management (5, 6). Nonetheless, negative effects on users’ physical and mental well-being have been reported due to extensive smartphone overuse. For example, overuse has been associated with anxiety and depression (7), loneliness (8), neck problems (9), interpersonal relationship problems (10), and accidents involving pedestrians and distracted drivers (11, 12). Smartphone overuse to the extent that it impacts one’s health and relationships has been referred to as smartphone addiction (13). Lin et al. (14) considered smartphone addiction a form of technological addiction, which Griffiths (15) defined as a non-chemical behavioral addiction involving interaction between human and machine.

Particular personality traits have been proposed among the key predictors of smartphone addiction. For example, the following have been associated with smartphone and internet addiction: low agreeableness and neuroticism (16–18), extraversion and openness to new experiences (18, 19), low conscientiousness and adjustment disorders (17), social interaction anxiety and locus of control issues (20), and narcissistic traits (8). Studies have investigated the relationship between family cohesion and smartphone addiction (10, 21, 22), but there is scarcity of research on self-cohesion and smartphone addiction.

### 1.1. Self-cohesion and smartphone addiction

The concept of self-cohesion originates from psychoanalyst Kohut (34) theory of the psychology of the self. This theory proposes two aspects of the self, one that is cohesive and another that is fragile (23, 24). Both refer to one’s healthy or unhealthy sense of self and self-esteem, and thereby well-being The development of a cohesive self from infancy to adulthood evolves along three axes: the grandiosity, idealization, and alter ego-connectedness axes (23–25). According to Kohut, the grandiosity axis concerns the ability of individuals to develop assertiveness and healthy ambitions and to maintain a positive sense of self-esteem (26). The idealization axis concerns the ability of individuals to possess and maintain healthy goal-setting (23). The alter ego-connectedness axis refers to the ability of individuals to form intimate relationships and express their feelings with significant others (25). Individuals with healthy development on the grandiosity, idealization, and alter ego-connectedness axes would be confident, would have healthy ambitions, goals, values, and ideals, and would feel a sense of connectedness with others (23, 25, 26).

Gleason (27) and Banai et al. (28) argued that the cohesive self can be considered a healthy form of narcissism, where individuals are able to feel pride in their accomplishments and in themselves. On the other hand, a lack of self-cohesion can manifest as negative narcissistic symptoms, including a sense of grandiosity and entitlement, wherein such individuals seek to compensate for their deficits in self-esteem with excessive self-promotion and self-presentation (29). Thus, it has been found that individuals with low self-cohesion tend to upload self-promoting and attractive pictures of themselves and update their social media status more frequently for the purpose of self-presentation (30–33). Gleason (27) further identified two dimensions of self-cohesion, presence and consistency. The presence dimension concerns individuals’ need for the presence of other people in order to enjoy a healthy sense of self and self-esteem. “A person with a fragile self may feel as though s/he does not exist unless in the presence of someone else. In other words, others are needed to provide the mirroring or idealizing functions to prevent the complete loss of self” [(27), p. 16]. The consistency dimension concerns individuals’ characteristic patterns of thought and behavior with regard to themselves and interactions with others. Low consistency individuals are “looking for others to perform selfobject functions, feel alienated from society, and have not successfully coordinated their self parts” [(27), p. 87]. On the other hand, a high consistency person is “more cohesive, having integrated the self parts, experiences less need for others to function as selfobjects and is therefore more likely to feel connected to the larger society” [(27), p. 87]. A number of studies have established a link between narcissistic traits and excessive smartphone use (34, 35). Nonetheless, despite the strong connection between narcissism and self-cohesion, no study to date has examined the association between self-cohesion and smartphone addiction. The first aim of this study was, therefore, to examine the association between self-cohesion and smartphone addiction. We hypothesized that self-cohesion is negatively related to smartphone addiction.

*Hypothesis 1*: Self-cohesion will be negatively associated with smartphone addiction.

### 1.2. The mediating role of rejection sensitivity

It has been postulated that the fear of being rejected is virtually universal (36, 37). However, some individuals are more concerned with the possibility of social rejection, the tendency that Kelly (38) referred to as rejection sensitivity. More concretely, rejection sensitivity (RS) represents one or more of the following: anticipatory anxiety over the possibility of social rejection, a tendency to expect rejection, or strong emotional reactions to actual rejection occurrences (39–41).

Rejection sensitivity has been related to both self-cohesion and smartphone addiction. Banai et al. (28) found that several personality traits of the low self-cohesive individual were positively related to rejection sensitivity. It can be assumed that high self-cohesion will negatively correlate with rejection sensitivity, that is, a person with high self-cohesion, who has a sense of connectedness with others (23, 26), would be less sensitive to rejection. Rejection sensitivity has also been related to smartphone and internet addiction. Sun et al. (42) reported a positive relationship between rejection sensitivity and smartphone addiction (42). A positive relationship was also found between rejection sensitivity and internet addiction by Fontana et al. (43) and Molavi et al. (44). And a study by Farahani et al. (45) showed that individuals with high rejection sensitivity spent more time using social media, in this particular case Facebook, than their counterparts.

Thus, this study’s second aim was to investigate the mediating role that rejection sensitivity might play in the relationship between self-cohesion and smartphone addiction, leading to the second hypothesis:

*Hypothesis 2*: Rejection sensitivity will negatively mediate the relationship between self-cohesion and smartphone addiction.

**Figure 1:**
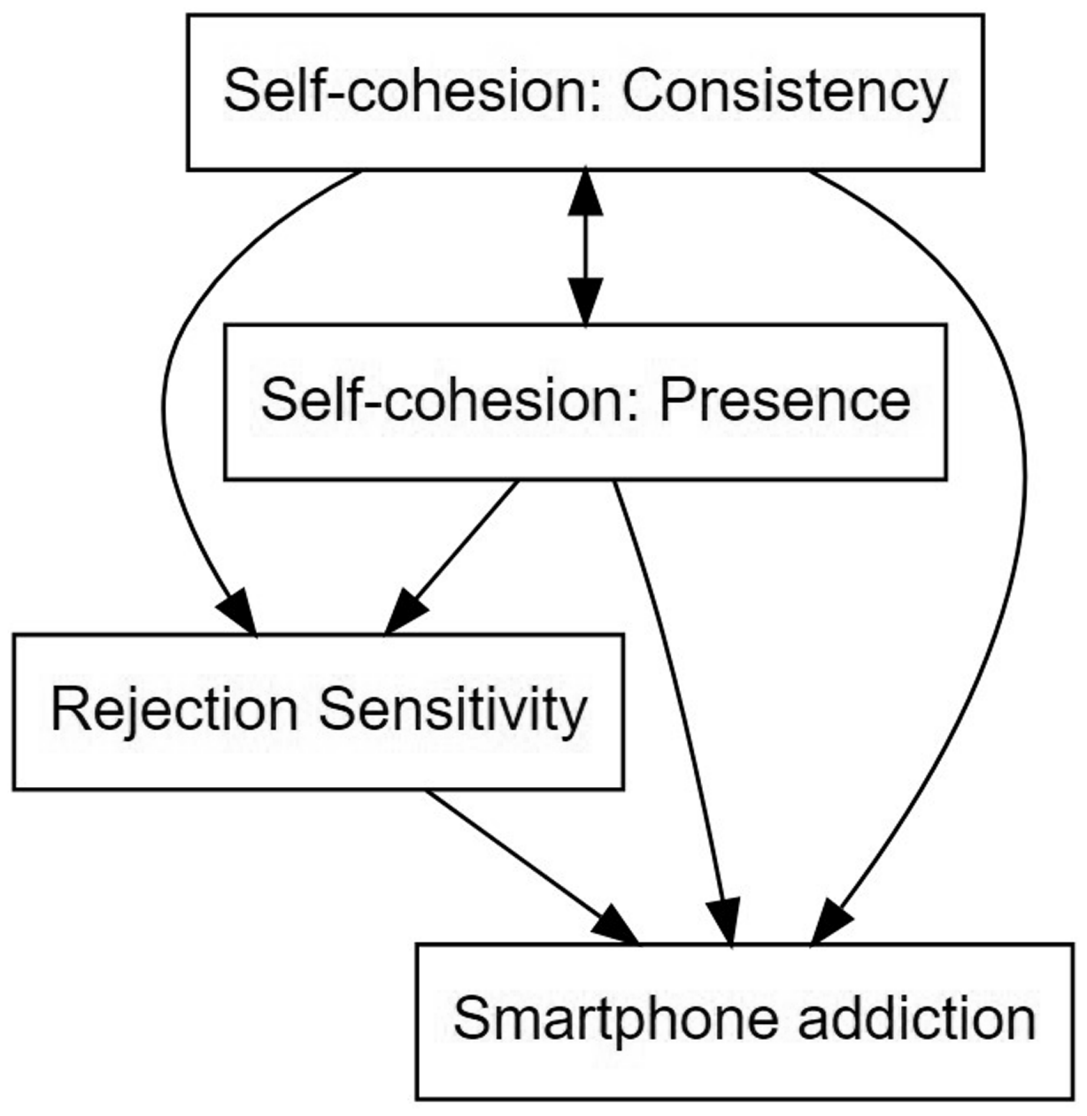
Conceptual framework.

The conceptual model guiding this study is represented in Figure 1.

## 2. Methods

### 2.1. Study design and sample

The study designer contacted the authors of the scales used in the study to obtain permission for their use in the study. Then, for those scales for which a validated Arabic version did not exist, the measures were translated into Arabic.

The study sample was composed of 910 participants, 58% females, with a mean age of 34.9 (SD = 13.6, range = 15–75). This sample was determined by convenience methods. Potential participants were invited via social media. The invitation informed them of the study’s aim and that their participation was voluntary and they could terminate their participation at any time. Confidentiality and anonymity were assured. Respondents provided informed consent. The questionnaire was sent to respondents via Facebook, email, and WhatsApp. Respondents were asked to be as honest as possible.

### 2.2. Measures

The questionnaire sent to participants contained a set of demographic questions, including gender, age, education, marital status, employment status, monthly income, time spent daily on a smartphone, and physical activity. This was followed by questions about frequency and duration of smartphone use, and the Self-Cohesion Scale (27), the Short Version of Smartphone Addiction Scale (46), and the Rejection Sensitivity RS-Adult Questionnaire (A-RSQ) (47).

#### 2.2.1. Self-cohesion scale

Self-Cohesion was assessed using the Self-Cohesion Scale (27), which was developed based on Kohut (34) theory of the psychology of the self. This scale contains 24 items and is scored on a six-point Likert scale ranging from 1 (strongly agree) to 6 (strongly disagree). Gleason (27) suggested a two-factor model for this scale, so exploratory factor analysis (EFA) was conducted on the scale prior to data analysis. The Bartlett test (*k* = 206.6, *df* = 24, *p* < 0.001) and the Kaiser-Meyer-Olkin test of sphericity (MSA = 0.89) indicated that the data were suitable for factor analysis. Nonetheless, the inspection of communalities showed that 14 items had communalities below 0.30 and were removed from the item pool. Those items were numbers 2, 4, 5, 6, 9, 14, 15, 16, 17, 19, 20, 21, 22, and 23. EFA was then conducted on the remaining 10 items (see Appendix). Consistent with Gleason (27), the scree plot indicated two factors that could be extracted, namely presence and consistency, as their respective items indicate. We see, therefore, that the conceptual model governing the study required the inclusion of these two separate dimensions of self-cohesion, as shown in Figure 1. The items in the presence and consistency dimensions had adequate factor loadings ranging between 0.330 and 0.782. In this study, 6 items measured the presence dimension and 4 items measured the consistency dimension. Possible scores range between 6 and 36 for presence and between 4 and 24 for consistency.

#### 2.2.2. Rejection sensitivity RS-adult questionnaire

Rejection sensitivity was assessed using the Rejection Sensitivity RS-Adult Questionnaire (A-RSQ) (47). This measure consists of nine items, each of which is scored on six-point Likert scale, ranging from 1 (“very unconcerned/very unlikely”) to 6 (“very concerned/very likely”). The scale consists of two components: concern over rejection and expectancy of acceptance. Respondents are presented nine imaginal situations involving interpersonal relationships, for example, “You approach a close friend to talk after doing or saying something that seriously upset him/her.” Respondents then answer two questions about each scenario, such as “How concerned or anxious would you be over whether or not your friend would want to talk with you?” indicating the respondent’s rejection concern, and “I would expect that he/she would want to talk with me to try to work things out,” indicating expectancy of acceptance. To form a composite score of rejection sensitivity, Berenson et al. (47) recommended multiplying the concern of rejection scores by the reverse of the corresponding expectancy of acceptance scores and then computing the average of the resulting scores. Possible scores range between 1 and 24.

#### 2.2.3. Short version of the smartphone addiction scale

Smartphone addiction was measured using the Short Version of the Smartphone Addiction Scale (46). This scale consists of 10 items, such as “I will not be able to stand not having a smartphone.” Respondents grade their reaction to each item on a six-point Likert scale, ranging from 1 (strongly disagree) to 6 (strongly agree). The overall scores range from 10 to 60, where higher scores indicate higher levels of smartphone addiction. This scale has proven psychometric properties (46). In this study, we used an Arabic version of the scale that has been validated by Al-Qarni and El Keshky (48).

### 2.3. Statistical analysis

The data analyses were conducted using RStudio (49), a statistical computing environment. We calculated the socio-demographic characteristics of the sample first. Then, exploratory factor analysis was conducted on the Self-Cohesion Scale (27) in order to verify the two factors suggested by Gleason’s original study. The EFA was computed using the “psych” software package (50). Descriptive statistics, Pearson correlations, and Cronbach’s alpha coefficients were then computed. The final stage was structural equation modeling using the “lavaan” software package (51). To plot the model, we used the “lavaanPlot” package (52).

### 2.4. Ethics

All procedures followed were in accordance with the ethical standards of the relevant institutional and national committees on human experimentation and with the Helsinki Declaration of 1975, as revised in 2000. Approval for conducting this study was obtained from the ethics committee of Institutional Review Board of King Abdulaziz University, Jeddah, Saudi Arabia, and informed consent was obtained from all subjects.

## 3. Results

The socio-demographic characteristics of the sample and the ANOVA tests are summarized in Table 1. Around 58% of the sample were female. With regard to education, 3.7% had less than high school, 15.4% had attended high school, 25% were college students, 43.2% had a university degree, and 12.7% held a master’s or doctoral degree. Around 40.3% were single, 55.5% were married, 3.1% divorced, and 1.1% were widowed. Thirty-six percent were employed full time, 3.5% were employed part time, 10.9% were retired, 28.3% were students, and 21.2% were unemployed. Around 11.2% were earning <3,000 RS per month, 10.4% were earning between 3,000 and 5,000 SR, 8.9% between 5,000 and 7,000 SR, 11.2% between 7,000 and 9,000 SR, 14.4% between 9,000 and 11,000 SR, and 43.9% were earning more than 11,000 SR per month. Only 3% of the sample spent <1 h per day using a smartphone, 20% were spending between 1 and 3 h, 40.2% between 4 and 6 h, 23% between 7 and 9 h, and 13.8% were spending more than 10 h per day using a smartphone. Sixty-five percent were living an active lifestyle and 35% were living a sedentary lifestyle.

**Table 1:** Socio-demographic characteristics and ANOVA tests.

Variable	*n*	%	SAS	RS	Presence	Consistency
**Gender**			*p* = 0.639	*p* < 0.05	*p* < 0.001	*p* = 0.101
Female	528	58	32.7 (9.01)	8.8 (3.57)	25.1 (7.14)	19.1 (4.51)
Male	382	42	32.4 (951)	8.3 (3.01)	26.74 (6.57)	18.5 (4.79)
**Education**			*p* < 0.05	*p* < 0.01	*p* < 0.001	*p* < 0.001
Less than high school	34	3.7	33.0 (6.76)	10.02 (5.28)	23.3 (7.00)	16.9 (4.05)
High School	140	15.4	34.2 (9.19)	8.3 (3.09)	25.9 (7.25)	18.2 (4.68)
College student	225	25	32.6 (9.72)	9.26 (3.54)	23.2 (6.67)	18.07 (5.15)
University degree	393	43.2	32.5 (9.42)	8.36 (3.07)	26.6 (6.75)	19.3 (4.37)
Master’s/doctoral degree	115	12.7	30.5 (7.80)	8.48 (3.28)	28.4 (6.09)	20.3 (3.94)
**Marital status**			*p* = 0.590	*p* < 0.001	*p* < 0.001	*p* < 0.001
Single	367	40.3	32.8 (9.62)	9.32 (3.58)	23.2 (6.80)	17.8 (5.09)
Married	505	55.5	32.3 (8.90)	8.18 (3.06)	27.6 (6.49)	19.5 (4.17)
Divorced	28	3.1	34.1 (9.31)	9.21 (4.03)	26.6 (5.76)	20.7 (3.69)
Widower	10	1.1	34.5 (10.45)	7.63 (2.78)	27.2 (8.58)	19.8 (4.31)
**Employment status**			*p* < 0.05	*p* < 0.01	*p* < 0.001	*p* < 0.001
Full-time	328	36	31.3 (9.61)	8.28 (2.93)	27.2 (6.39)	19.5 (4.32)
Part-time	32	3.5	32.1 (8.87)	8.70 (3.09)	26.4 (5.31)	19.4 (4.48)
Retired	99	10.9	33.4 (8.45)	8.05 (2.75)	30.2 (4.87)	19.8 (4.11)
Student	258	28.3	33.0 (9.45)	9.32 (3.76)	23.2 (6.87)	17.8 (5.10)
Unemployed	191	21.2	33.6 (8.50)	8.77 (3.63)	24.5 (7.83)	18.5 (4.53)
**Income per month, in SR**			*p* = 9.54	*p* < 0.01	*p* < 0.001	*p* < 0.01
<3,000	102	11.2	32.1 (8.50)	9.30 (3.65)	23.3 (7.11)	18.00 (4.98)
3,000 to <5,000	95	10.4	31.7 (9.15)	8.78 (3.27)	24.7 (6.89)	19.4 (4.34)
5,000 to <7,000	81	8.9	33.9 (9.46)	9.46 (3.48)	23.7 (5.86)	17.3 (5.13)
7,000 to <9,000	102	11.2	32.7 (9.04)	9.09 (3.43)	26.4 (6.63)	18.2 (4.78)
9,000 to <11,000	131	14.4	33.2 (9.43)	8.15 (2.87)	26.05 (6.99)	18.9 (4.30)
≥11,000	399	43.9	32.3 (9.35)	8.38 (3.34)	26.9 (6.94)	19.4 (4.48)
**Hours per day using smartphone**			*p* < 0.001	*p* < 0.05	*p* < 0.001	*p* < 0.01
<1 h	27	3	28.9 (6.56)	8.33 (2.26)	24.5 (7.28)	19.4 (4.73)
1–3 h	182	20	28.6 (7.62)	8.08 (3.43)	27.5 (6.64)	19.8 (4.10)
4–6 h	365	40.2	31.4 (8.98)	8.57 (3.23)	26.4 (6.96)	18.8 (4.63)
7–9 h	209	23	35.0 (9.10)	9.13 (3.06)	25.03 (6.91)	18.8 (4.54)
≥10 h	126	13.8	38.1 (9.08)	9.15 (4.03)	23.07 (6.37)	17.6 (5.22)
**Physical activity**			*p* < 0.001	*p* = 0.193	*p* < 0.05	*p* = 0.669
Active	591	65	31.6 (8.96)	8.56 (3.18)	26.1 (6.74)	18.9 (4.57)
Sedentary	319	35	34.2 (9.46)	8.86 (3.64)	25.1 (7.27)	18.7 (4.76)

SAS, Smartphone Addiction Scale score; RS, rejection sensitivity score on A-RSQ; all scores shown as Mean (SD).

In terms of differences based on demographics, there were no gender differences in smartphone addiction scores, but females had higher scores on rejection sensitivity and males had higher scores on presence. In terms of education, those who had a high school education reported the highest scores of smartphone addiction, those with less than high school education had higher scores of rejection sensitivity, and those with a master’s or doctoral degree had significantly higher scores on the presence and consistency dimensions of self-cohesion. Regarding marital status, single respondents had higher scores on rejection sensitivity, married individuals had higher scores on presence, and divorced respondents had higher scores on consistency. Unemployed individuals had higher scores on smartphone addiction, students had higher scores of rejection sensitivity, those working full-time had higher scores on the presence dimension, and retired individuals had higher scores on the presence and consistency dimensions. In terms of income, those earning at least 11,000 SR had significantly higher scores on the presence and consistency dimensions of self-cohesion. As expected, those who were spending more than 10 h per day using a smartphone reported higher scores on smartphone addiction, and they also scored higher on rejection sensitivity. Those who spent between one and 3 h using a smartphone reported higher scores on the presence and consistency dimensions. Finally, repsondents living a sedentary lifestyle scored higher on smartphone addiction and those who were active reported higher scores on the presence dimension of self-cohesion.

The descriptive statistics of the study variables, Pearson correlations, and Cronbach’s alpha coefficients are displayed in Table 2. The mean score for smartphone addiction was 32.6 (SD = 9.22, range = 6–60), the average score for rejection sensitivity was 8.67 (SD = 3.35, range = 1–24), the average score for presence was 25.8 (SD = 6.95, range = 6–36), and the mean score for consistency was 18.9 (SD = 4.46, range = 6–24). Smartphone addiction was positively correlated with rejection sensitivity (*r* = 0.19, *p* < 0.001). On the other hand, smartphone addiction was negatively correlated with presence (*r* = −0.29, *p* < 0.001) and consistency (*r* = −0.28, *p* < 0.001). As expected, rejection sensitivity was also negatively correlated with both presence (*r* = −0.39, *p* < 0.001) and consistency (*r* = −0.32, *p* < 0.001). The internal consistency reliability was adequate for smartphone addiction (α = 0.80), for rejection sensitivity (α = 0.72), for presence (α = 0.80), and for consistency (α = 0.78).

**Table 2:** Pearson correlations between the variables and Cronbach’s alpha coefficients.

Variable	Mean (SD)	Smartphone addiction	Rejection sensitivity	Presence	Consistency	Alpha
Smartphone addiction	32.6 (9.22)	1				0.80
Rejection sensitivity	8.67 (3.35)	0.19***	1			0.72
Presence	25.8 (6.95)	−0.29***	−0.39***	1		0.80
Consistency	18.9 (4.64)	−0.28***	−0.32***	0.61***	1	0.78

***p < 0.001.

**Figure 2:**
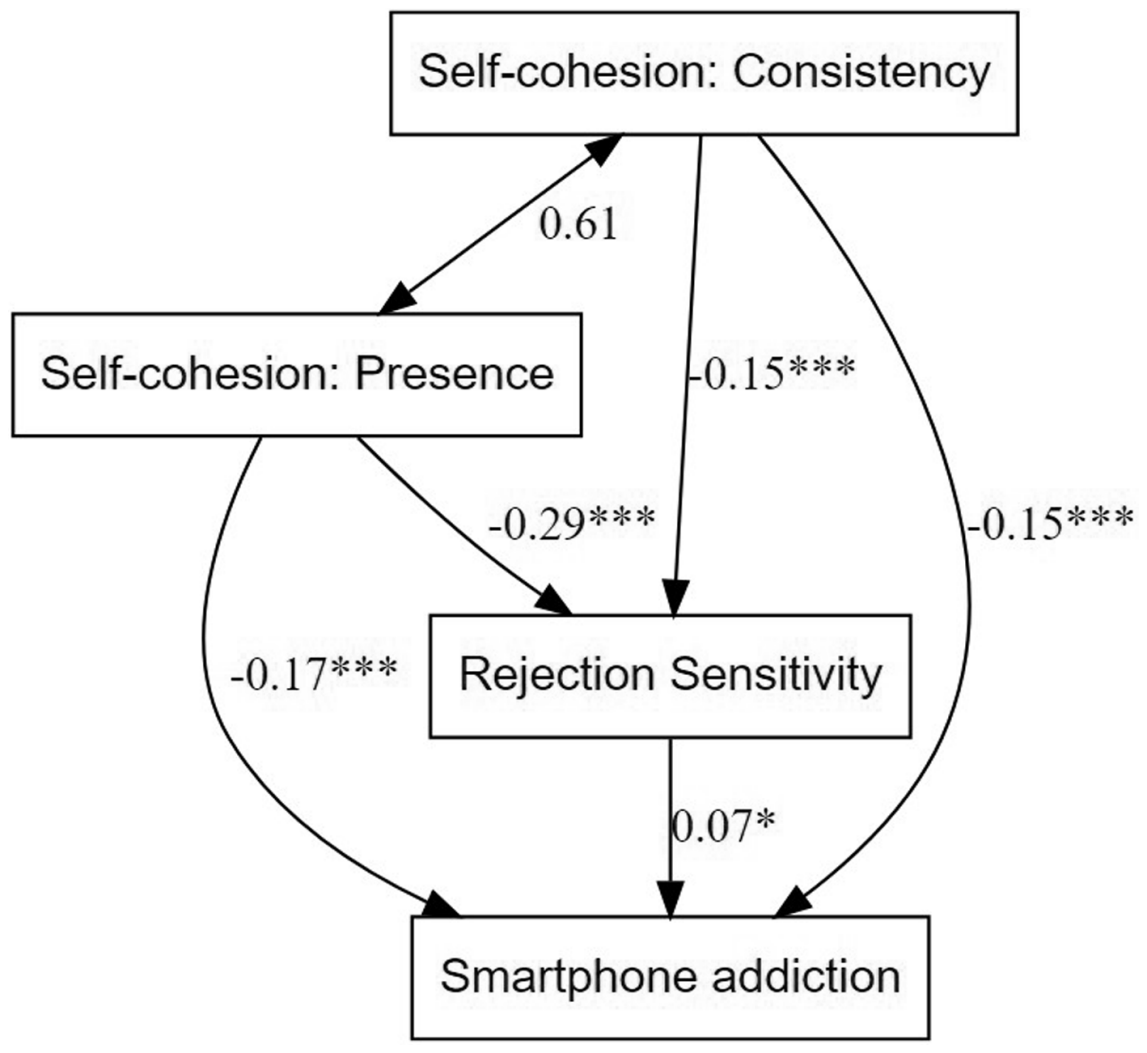
Structural equation model predicting smartphone addiction. *p<0.05; ***p<0.001; Numbers on arrows represent the standardized regression coefficients, and the number on the double-sided arrow represents the covariance.

In the structural equation model, the self-cohesion presence component negatively predicted smartphone addiction (*β* = −0.17, *p* < 0.001), as did self-cohesion consistency (*β* = −0.15, *p* < 0.001). Rejection sensitivity negatively mediated the relationship between presence and smartphone addiction (β_ind_ = −0.028, *p* < 0.05). Rejection sensitivity also negatively mediated the relationship between consistency and smartphone addiction (β_ind_ = −0.022, *p* < 0.05). This model exhibited good model fit (*ꭓ*^2^ = 256.90, *p* < 0.001; RMSEA = 0.02; SRMR = 0.01; CFI = 0.99; TLI = 0.98; Figure 2).

## 4. Discussion

Given the continuing increase in smartphone usage, with appealing applications that may increase the risk of smartphone addiction, there is a need to investigate the antecedents of smartphone addiction and the pathways through which these relationships operate. Accordingly, this study examined the relationship between self-cohesion – in particular, its consistency and presence dimensions – and smartphone addiction, as well as the mediation role of rejection sensitivity. As expected, the results indicated that the presence and consistency dimensions of self-cohesion negatively predicted smartphone addiction and this relationship was negatively mediated by rejection sensitivity. Thus, the results supported *Hypotheses 1* and *2*.

Cohesive individuals possess an integrated and healthy sense of self, which may help to explain their negative association with smartphone addiction. Non-cohesive individuals have a fragile sense of self and are ill at ease unless they are the presence of other people (27). The latter are at higher risk of smartphone addiction perhaps because the need for the presence of others is satisfied by their digital “presence.” Further, in order to compensate for their needs, such individuals may exhibit grandiose, self-promotional, and self-presenting behaviors, which have been associated with the risk of smartphone addiction (34, 35, 53, 54). A study by Pearson and Hussain (54) indeed reported a positive relationship between narcissism and smartphone addiction. Similarly, Ksinan et al. (35) found that grandiosity was related to compulsive smartphone use. A study by Gosling et al. (53) showed that individuals who scored high on narcissism were more likely to spend more time on social media. Andreassen et al. (34) reported in survey of social media users that narcissistic individuals, who need to feed their ego, tended to be addicted to social media. This evidence demonstrates that individuals with low self-cohesion tend to be addicted to smartphones.

The findings of this study demonstrated that rejection sensitivity mediates the relationship between self-cohesion and smartphone addiction. This corroborates prior research. Rejection sensitivity has been related to self-cohesion (28) and smartphone addiction (42). Interestingly, the literature has established rejection sensitivity as a mediating variable between a range of other variables and smartphone or internet addiction. For example, rejection sensitivity was found to mediate the relationship between attachment style and smartphone addiction (55). Another study reported that the relationship between online self-disclosure and internet addiction was mediated by rejection sensitivity. And Xin et al. (56) found that rejection sensitivity mediated the relationship between cyber-victimization and internet addiction.

People with high rejection sensitivity may be prone to social media addiction because they create for themselves an environment online in which they interact with others without feeling uncomfortable, as they might be in face-to-face interaction (57). It has also been postulated that people engage in excessive internet or smartphone use as a coping mechanism to regulate emotions following a rejection stress event (44, 58, 59).

This study found no gender differences in smartphone addiction, which corroborates the findings of Andrade et al. (60). Nonetheless, others have reported that females were more likely to be addicted than males (17, 61). We found that females were more likely to be sensitive to rejection, which is in line with other studies (62). Individuals with lower levels of education had higher smartphone addiction and rejection sensitivity scores, which is in line with the results from Kwon et al. (13). However, Hawes et al. (63) reported higher rejection sensitivity in college students compared to high school students. This study also found differences in smartphone addiction and rejection sensitivity based on marital status and employment status. This has also been reported in previous studies (64–67). We further found that smartphone addiction scores were higher in individuals living a sedentary lifestyle and this was also reported by Manzoor et al. (68). Finally, those who spent more hours using a smartphone had higher scores not only on smartphone addiction, as might be expected, but also on rejection sensitivity. The former finding is in line with previous research that demonstrated that the severity of smartphone addiction is associated with duration and frequency of smartphone use (69, 70).

### 4.1. Implications of the study

It is not possible to control the increasing rate of smartphone usage in the world. Thus, it would be wise to put effort into ameliorating the antecedent or predictor factors of smartphone addiction. The findings of this study suggest creating programs and training to improve individuals’ self-cohesion and reduce their rejection sensitivity, especially in young people. This is critical for parents, educators, and counselors to reduce the negative consequences of excessive smartphone usage.

### 4.2. Limitations

Despite the strengths of this study, there are some limitations that should be acknowledged. First, the design of the study was cross-sectional, which does not allow conclusions with regard to causation. Second, the sample was determined using convenience methods. Future research should use random sampling methods. Third, 14 items of the Self-Cohesion Scale were not used in this study because of their low communalities.

## 5. Conclusion

This study contributes to the literature by having examined and established the association between self-cohesion and smartphone addiction, and by confirming the mediation role of rejection sensitivity. The findings indicate that self-cohesion’s dimensions of presence and consistency are predictors of smartphone addiction, and rejection sensitivity is a mediator of these relationships. These findings add new insights, as the contribution of self-cohesion to smartphone addiction had not previously been investigated. It appears that increased self-cohesion and decreased rejection sensitivity could be foci for interventions to deal with smartphone addiction. These findings yield practice insights for counselors, educators, parents, and policy makers, who can use these findings to motivate and shape the design of programs to counteract smartphone addiction.

## Data availability statement

The raw data supporting the conclusions of this article will be made available by the corresponding author, without undue reservation.

## Ethics statement

The studies involving human participants were reviewed and approved by the Ethics Committee of Institutional Review Board of the King Abdulaziz University, Jeddah, Saudi Arabia. The participants provided their written informed consent to participate in this study.

## Author contributions

ME contributed to the definition of research objectives, model and hypotheses and funding acquisition. ME and YS participated in data analysis plan, writing—original draft, and approval of the final manuscript. ME, HA, AA, and YS contributed to the provision of materials (i.e., questionnaires), participated in data collection, review and editing. All authors have read and approved the final version of the manuscript.

## Funding

This research work was funded by the Institutional Fund Projects under grant no. (IFPIP: 246-246-1443).

## Conflict of interest

The authors declare that the research was conducted in the absence of any commercial or financial relationships that could be construed as a potential conflict of interest.

## Publisher’s note

All claims expressed in this article are solely those of the authors and do not necessarily represent those of their affiliated organizations, or those of the publisher, the editors and the reviewers. Any product that may be evaluated in this article, or claim that may be made by its manufacturer, is not guaranteed or endorsed by the publisher.

## References

[1] Statista. (2023). Number of Smartphone Subscriptions Worldwide From 2016 to 2022, with Forecasts from 2023 to 2027. Available at: https://www.statista.com/statistics/330695/number-of-smartphone-users-worldwide/ (Accessed January 13, 2023).

[2] Statista. (2023). Number of Smartphone Users in Saudi Arabia From 2017 to 2025. Available at: https://www.statista.com/statistics/494616/smartphone-users-in-saudi-arabia/ (Accessed 13 January 2023).

[3] Haug S Paz Castro R Kwon M Filler A Kowatsch T Schaub MP. Smartphone use and smartphone addiction among young people in Switzerland. J Behav Addict. (2015) 4:299–307. doi: 10.1556/2006.4.2015.037, PMID: 26690625 PMC4712764

[4] Gustafson DH McTavish FM Chih MY Atwood AK Johnson RA Boyle MG. A smartphone application to support recovery from alcoholism a randomized clinical trial. JAMA Psychiat. (2014) 71:566–72. doi: 10.1001/jamapsychiatry.2013.4642, PMID: 24671165 PMC4016167

[5] Arsand E Muzny M Bradway M Muzik J Hartvigsen G. Performance of the first combined smartwatch and smartphone diabetes diary application study. J Diabetes Sci Technol. (2015) 9:556–63. doi: 10.1177/1932296814567708, PMID: 25591859 PMC4604524

[6] Bain TM Jones ML O’Brian CA Lipman R. Feasibility of smartphone-delivered diabetes self-management education and training in an underserved urban population of adults. J Telemed Telecare. (2015) 21:58–60. doi: 10.1177/1357633X14545426 25059245

[7] Elhai JD Dvorak RD Levine JC Hall BJ. Problematic smartphone use: a conceptual overview and systematic review of relations with anxiety and depression psychopathology. J Affect Disord. (2017) 207:251–9. doi: 10.1016/j.jad.2016.08.030, PMID: 27736736

[8] Gökçearslan Ş Yildiz Durak H Berikan B Saritepeci M. Smartphone addiction, loneliness, narcissistic personality, and family belonging among university students: a path analysis. Soc Sci Q. (2021) 102:1743–60. doi: 10.1111/ssqu.12949

[9] Lee S Kang H Shin G. Head flexion angle while using a smartphone. Ergonomics. (2015) 58:220–6. doi: 10.1080/00140139.2014.967311, PMID: 25323467

[10] Hawi NS Samaha M. Relationships among smartphone addiction, anxiety, and family relations. Behav Inf Technol. (2017) 36:1046–52. doi: 10.1080/0144929X.2017.1336254

[11] Klauer SG Guo F Simons-Morton BG Ouimet MC Lee SE Dingus TA. Distracted driving and risk of road crashes among novice and experienced drivers. N Engl J Med. (2014) 370:54–9. doi: 10.1056/nejmsa1204142, PMID: 24382065 PMC4183154

[12] Shelton JT Elliott EM Eaves SD Exner AL. The distracting effects of a ringing cell phone: an investigation of the laboratory and the classroom setting. J Environ Psychol. (2009) 29:513–21. doi: 10.1016/j.jenvp.2009.03.001, PMID: 21234286 PMC3018855

[13] Kwon M Lee JY Won WY Park JW Min JA Hahn C. Development and validation of a smartphone addiction scale (SAS). PLoS One. (2013) 8:e56936. doi: 10.1371/journal.pone.0056936, PMID: 23468893 PMC3584150

[14] Lin YH Chang LR Lee YH Tseng HW Kuo TBJ Chen SH. Development and validation of the smartphone addiction inventory (SPAI). PLoS One. (2014) 9:e98312. doi: 10.1371/journal.pone.0098312, PMID: 24896252 PMC4045675

[15] Griffiths M. Gambling on the internet: a brief note. J Gambl Stud. (1996) 12:471–3. doi: 10.1007/BF01539190, PMID: 24234164

[16] Kuss DJ Shorter GW Van Rooij AJ Van De Mheen D Griffiths MD. The internet addiction components model and personality: establishing construct validity via a nomological network. Comput Hum Behav. (2014) 39:312–21. doi: 10.1016/j.chb.2014.07.031

[17] Müller KW Wölfling K Beutel ME Stark B Quiring O Aufenanger S. Insights into aspects behind internet-related disorders in adolescents: the interplay of personality and symptoms of adjustment disorders. J Adolesc Health. (2018) 62:234–40. doi: 10.1016/j.jadohealth.2017.09.011, PMID: 29174875

[18] Zhou Y Li D Li X Wang Y Zhao L. Big five personality and adolescent internet addiction: the mediating role of coping style. Addict Behav. (2017) 64:42–8. doi: 10.1016/j.addbeh.2016.08.009, PMID: 27543833

[19] Servidio R. Exploring the effects of demographic factors, internet usage and personality traits on internet addiction in a sample of Italian university students. Comput Hum Behav. (2014) 35:85–92. doi: 10.1016/j.chb.2014.02.024

[20] Lee YK Chang CT Lin Y Cheng ZH. The dark side of smartphone usage: psychological traits, compulsive behavior and technostress. Comput Hum Behav. (2014) 31:373–83. doi: 10.1016/j.chb.2013.10.047

[21] Jimeno MV Ricarte JJ Toledano A Mangialavori S Cacioppo M Ros L. Role of attachment and family functioning in problematic smartphone use in young adults. J Fam Issues. (2022) 43:375–91. doi: 10.1177/0192513X21993881

[22] Rachmat IF Hartati S Erdawati E. Family cohesion, interpersonal communication, and smartphone addiction: does it affect children’s emotional dysregulation? Cakrawala Pendidikan. (2021) 40:279–91. doi: 10.21831/cp.v40i2.34214

[23] Kohut H. The Analysis of the Self International University Press (1971).

[24] Kohut H. The Restoration of the Self. New York: International Universities Press, Inc. (1977).

[25] Kohut Heinz. (1984). How Does Analysis Cure?. Chicago: University of Chicago Press, 6, 403–428

[26] Kohut H. The psychoanalytic treatment of narcissistic personality disorders: outline of a systematic approach. Psychoanal Study Child. (1968) 23:86–113. doi: 10.1080/00797308.1968.11822951, PMID: 5759031

[27] Gleason DK. The Self Cohesion Scale: A Measure of the Kohutian Concept of Self Cohesion. Knoxville: University of Tennessee (2005).

[28] Banai E Mikulincer M Shaver PR. “Selfobject” needs in Kohut’s self psychology: links with attachment, self-cohesion, affect regulation, and adjustment. Psychoanal Psychol. (2005) 22:224–60. doi: 10.1037/0736-9735.22.2.224

[29] Miller IJ. Interpersonal vulnerability and narcissism: a conceptual continuum for understanding and treating narcissistic psychopathology. Psychotherapy. (1992) 29:216–24. doi: 10.1037/0033-3204.29.2.216

[30] Buffardi LE Campbell WK. Narcissism and social networking web sites. Personal Soc Psychol Bull. (2008) 34:1303–14. doi: 10.1177/0146167208320061, PMID: 18599659

[31] McKinney BC Kelly L Duran RL. Narcissism or openness?: college students’ use of Facebook and twitter. Commun Res Rep. (2012) 29:108–18. doi: 10.1080/08824096.2012.666919

[32] Sorokowski P Sorokowska A Oleszkiewicz A Frackowiak T Huk A Pisanski K. Selfie posting behaviors are associated with narcissism among men. Personal Individ Differ. (2015) 85:123–7. doi: 10.1016/j.paid.2015.05.004

[33] Wang JL Jackson LA Zhang DJ Su ZQ. The relationships among the big five personality factors, self-esteem, narcissism, and sensation-seeking to Chinese university students’ uses of social networking sites (SNSs). Comput Hum Behav. (2012) 28:2313–9. doi: 10.1016/j.chb.2012.07.001

[34] Andreassen CS Pallesen S Griffiths MD. The relationship between addictive use of social media, narcissism, and self-esteem: findings from a large national survey. Addict Behav. (2017) 64:287–93. doi: 10.1016/j.addbeh.2016.03.006, PMID: 27072491

[35] Ksinan AJ Mališ J Vazsonyi AT. Swiping away the moments that make up a dull day: narcissism, boredom, and compulsive smartphone use. Curr Psychol. (2021) 40:2917–26. doi: 10.1007/s12144-019-00228-7

[36] Baumeister RF Leary MR. The need to belong: desire for interpersonal attachments as a fundamental human motivation. Psychol Bull. (1995) 117:497–529. doi: 10.1037/0033-2909.117.3.497, PMID: 7777651

[37] Noelle-neumann E. The spiral of silence. J Commun. (1974) 24:43–51. doi: 10.1111/j.1460-2466.1974.tb00367.x

[38] Kelly K. Individual differences in reactions to rejection In: Leary MR, editor. Interpersonal Rejection. New York: Oxford University Press (2001). 291–315.

[39] Demircioğlu ZI Göncü-Köse A. Antecedents of problematic social media use and cyberbullying among adolescents: attachment, the dark triad and rejection sensitivity. Curr Psychol. (2022):1–19. doi: 10.1007/s12144-022-04127-2, PMID: 36540693 PMC9754995

[40] Downey G Feldman SI. Implications of rejection sensitivity for intimate relationships. J Pers Soc Psychol. (1996) 70:1327–43. doi: 10.1037/0022-3514.70.6.1327, PMID: 8667172

[41] Mehrabian A. The development and validation of measures of affiliative tendency and sensitivity to rejection. Educ Psychol Meas. (1970) 30:417–28. doi: 10.1177/001316447003000226

[42] Sun X Zhang Y Niu G Tian Y Xu L Duan C. Ostracism and problematic smartphone use: the mediating effect of social self-efficacy and moderating effect of rejection sensitivity. Int J Ment Heal Addict. (2021):1–20. doi: 10.1007/s11469-021-00661-5

[43] Fontana A Callea A Casini E Curti V. Rejection sensitivity and internet addiction in adolescence: exploring the mediating role of emerging personality disorders. Clin Neuropsychiatry. (2018) 15:206–14.

[44] Molavi P Mikaeili N Ghaseminejad MA Kazemi Z Pourdonya M. Social anxiety and benign and toxic online self-disclosures an investigation into the role of rejection sensitivity, self-regulation, and internet addiction in college students. J Nerv Ment Dis. (2018) 206:598–605. doi: 10.1097/NMD.0000000000000855, PMID: 30020206

[45] Farahani HA Aghamohamadi S Kazemi Z Bakhtiarvand F Ansari M. Examining the relationship between sensitivity to rejection and using Facebook in university students. Procedia Soc Behav Sci. (2011) 28:807–10. doi: 10.1016/j.sbspro.2011.11.147

[46] Kwon M Kim DJ Cho H Yang S. The smartphone addiction scale: development and validation of a short version for adolescents. PLoS One. (2013) 8:1–7. doi: 10.1371/journal.pone.0083558, PMID: 24391787 PMC3877074

[47] Berenson KR Gyurak A Ayduk Ö Downey G Garner MJ Mogg K. Rejection sensitivity and disruption of attention by social threat cues. J Res Pers. (2009) 43:1064–72. doi: 10.1016/j.jrp.2009.07.007, PMID: 20160869 PMC2771869

[48] Al-Qarni MS El Keshky ME. Psychometric properties of the Arabic short version of the smartphone addiction scale (SAS-SV) in Saudi Arabia. JKAU Arts Humanit. (2022) 30:400–14.

[49] Rstudio Team. RStudio: Integrated Development Environment for R. Boston, MA: RStudio, PBC (2022).

[50] Revelle W. (2017). Using the Psych Package to Generate and Test Structural Models. Available at: https://personality-project.org/r/psych_for_sem.pdf (Accessed December 25, 2022).

[51] Rosseel Y. Lavaan: an R package for structural equation modeling. J Stat Softw. (2012) 48:2–4. doi: 10.18637/jss.v048.i02

[52] Lishinski A. (2020). lavaanPlot 0.5.1. Available at: https://www.alexlishinski.com/post/lavaanplot-0-5-1/ (Accessed December 25, 2022).

[53] Gosling SD Augustine AA Vazire S Holtzman N Gaddis S. Manifestations of personality in online social networks: self-reported facebook-related behaviors and observable profile information. Cyberpsychol Behav Soc Netw. (2011) 14:483–8. doi: 10.1089/cyber.2010.0087, PMID: 21254929 PMC3180765

[54] Pearson C Hussain Z. Smartphone use, addiction, narcissism, and personality: a mixed methods investigation. Int J Cyber Behav Psychol Learn. (2015) 5:17–32. doi: 10.4018/ijcbpl.2015010102

[55] Hassani B Pirklani RK Ahadi B. Attachment styles and smartphone addiction: the mediating role of self-esteem and sensitivity to rejection. Mon J Psychol Sci. (2022) 21:971–86. doi: 10.52547/JPS.21.113.971

[56] Xin M Chen P Liang Q Yu C Zhen S Zhang W. Cybervictimization and adolescent internet addiction: a moderated mediation model. Int J Environ Res Public Health. (2021) 18:1–13. doi: 10.3390/ijerph18052427, PMID: 33801345 PMC7967556

[57] Demircioğlu ZI Göncü Köse A. Effects of attachment styles, dark triad, rejection sensitivity, and relationship satisfaction on social media addiction: a mediated model. Curr Psychol. (2021) 40:414–28. doi: 10.1007/s12144-018-9956-x

[58] Caplan SE. Relations among loneliness, social anxiety, and problematic internet use. CyberPsychol Behav. (2007) 10:234–42. doi: 10.1089/cpb.2006.9963, PMID: 17474841

[59] Weinstein A Abu HB Timor A Mama Y. Delay discounting, risk-taking, and rejection sensitivity among individuals with internet and video gaming disorders. J Behav Addict. (2016) 5:674–82. doi: 10.1556/2006.5.2016.081, PMID: 27958761 PMC5370373

[60] Andrade ALM Scatena A Martins GDG Pinheiro BDO Becker da Silva A Enes CC. Validation of smartphone addiction scale – short version (SAS-SV) in Brazilian adolescents. Addict Behav. (2020) 110:106540. doi: 10.1016/j.addbeh.2020.106540, PMID: 32682269

[61] Cheung T Lee RLT Tse ACY Do CW So BCL Szeto GPY. Psychometric properties and demographic correlates of the smartphone addiction scale-short version among Chinese children and adolescents in Hong Kong. Cyberpsychol Behav Soc Netw. (2019) 22:714–23. doi: 10.1089/cyber.2019.0325, PMID: 31621411

[62] Beeson CML Brittain H Vaillancourt T. The temporal precedence of peer rejection, rejection sensitivity, depression, and aggression across adolescence. Child Psychiatry Hum Dev. (2020) 51:781–91. doi: 10.1007/s10578-020-01008-2, PMID: 32462359

[63] Hawes T Zimmer-Gembeck MJ Campbell SM. Unique associations of social media use and online appearance preoccupation with depression, anxiety, and appearance rejection sensitivity. Body Image. (2020) 33:66–76. doi: 10.1016/j.bodyim.2020.02.010, PMID: 32113009

[64] Luterek JA Harb GC Heimberg RG Marx BP. Interpersonal rejection sensitivity in childhood sexual abuse survivors: mediator of depressive symptoms and anger suppression. J Interpers Violence. (2004) 19:90–107. doi: 10.1177/0886260503259052, PMID: 14680531

[65] Pachankis JE Goldfried MR Ramrattan ME. Extension of the rejection sensitivity construct to the interpersonal functioning of gay men. J Consult Clin Psychol. (2008) 76:306–17. doi: 10.1037/0022-006X.76.2.306, PMID: 18377126

[66] Ratan ZA Parrish AM Alotaibi MS Hosseinzadeh H. Prevalence of smartphone addiction and its association with sociodemographic, physical and mental well-being: a cross-sectional study among the young adults of Bangladesh. Int J Environ Res Public Health. (2022) 19:16583. doi: 10.3390/ijerph192416583, PMID: 36554468 PMC9778917

[67] Zhong Y Ma H Liang YF Liao CJ Zhang CC Jiang WJ. Prevalence of smartphone addiction among Asian medical students: a meta-analysis of multinational observational studies. Int J Soc Psychiatry. (2022) 68:1171–83. doi: 10.1177/00207640221089535, PMID: 35422151

[68] Manzoor A Basri R Ali I Javaid S Amjad M Amin U. Smartphone addiction/ overuse and its effect on dietary behavior and lifestyle – a systematic review. EAS J Nut Food Sci. (2020) 2:289–97. doi: 10.36349/easjnfs.2020.v02i05.007

[69] Lee H Ahn H Choi S Choi W. The SAMS: smartphone addiction management system and verification. J Med Syst. (2014) 38:1. doi: 10.1007/s10916-013-0001-1 24395031

[70] Lin YH Lin YC Lee YH Lin PH Lin SH Chang LR. Time distortion associated with smartphone addiction: identifying smartphone addiction via a mobile application (app). J Psychiatr Res. (2015) 65:139–45. doi: 10.1016/j.jpsychires.2015.04.003, PMID: 25935253

