# Corrigendum: The relationship between self-cohesion and smartphone addiction: the mediating role of rejection sensitivity

**DOI:** 10.3389/fpubh.2023.1257600

**Published:** 2023-09-04

**Authors:** Mogeda El Sayed El Keshky, Huda Aseem, Amira Alzain, Yasser Abdelazim Abdelmawgoud Samak

**Affiliations:** ^1^Department of Psychology, Faculty of Arts and Humanities, King Abdulaziz University, Jeddah, Saudi Arabia; ^2^Department of Geography and GIS, Faculty of Arts, Assiut University, Assiut, Egypt

**Keywords:** smartphone addiction, self-cohesion, rejection sensitivity, mediation analysis, Saudi Arabia

In the published article, there was an error in the author list, and author [Yasser Abdelazim Abdelmawgoud Samak] was erroneously [excluded]. The corrected authors list and affiliations are appears below.

Mogeda El Sayed El Keshky^1*^, Huda Aseem^1^, Amira Alzain^1^ and Yasser Abdelazim Abdelmawgoud Samak^2^

^1^Department of Psychology, Faculty of Arts and Humanities, King Abdulaziz University, Jeddah, Saudi Arabia

^2^Department of Geography and GIS, Faculty of Arts, Assiut University, Assiut, Egypt.

In the published article, there was an error in the Author contributions. The statement was incorrectly written as “MK contributed to the definition of research objectives, model and hypotheses, data analysis plan, writing – original draft, funding acquisition, and reviewing and approval of the final manuscript. HA and AA contributed to the provision of materials (i.e., questionnaires), participated in data collection. All authors contributed to the article and approved the submitted version.”

**The corrected statement is:**

ME contributed to the definition of research objectives, model and hypotheses and funding acquisition. ME and YS participated in data analysis plan, writing—original draft, and approval of the final manuscript. ME, HA, AA, and YS contributed to the provision of materials (i.e., questionnaires), participated in data collection, review and editing. All authors have read and approved the final version of the manuscript.

The authors apologize for this error and state that this does not change the scientific conclusions of the article in any way. The original article has been updated.

