# Supplementary material for: The relationship between self-cohesion and smartphone addiction: the mediating role of rejection sensitivity
Source: Front Public Health. 2023 Jun 28;11:1166864. doi: 10.3389/fpubh.2023.1166864 (PMC10338100; doi:10.3389/fpubh.2023.1166864)
Supplement: Supplementary file 1 [file Table_1.docx]

**Appendix**

| **EFA factor loadings for the Self-Cohesion Scale** | | | | |
| --- | --- | --- | --- | --- |
|  | **Presence** | | **Consistency** | |
|  | Communalities | Factor loadings | Communalities | Factor loadings |
| Item 1 | 0.35 | 0.69 |  |  |
| **item 2** | **0.23** | **0.48** |  |  |
| Item 3 |  |  | 0.35 | 0.78 |
| **Item 4** |  |  | **0.13** | **0.36** |
| **Item 5** |  |  | **0.11** | **0.34** |
| **Item 6** | **0.06** | **0.26** |  |  |
| Item 7 | 0.34 | 0.67 |  |  |
| Item 8 | 0.43 | 0.65 |  |  |
| **Item 9** |  |  | **0.01** | **0.11** |
| Item 10 |  |  | 0.36 | 0.62 |
| Item 11 | 0.37 | 0.44 |  |  |
| Item 12 |  |  | 0.36 | 0.72 |
| Item 13 | 0.32 | 0.33 |  |  |
| **Item 14** | **0.27** | **0.52** |  |  |
| **Item 15** | **0.26** | **0.51** |  |  |
| **Item 16** |  |  | **0.06** | **0.23** |
| **Item 17** | **0.17** | **0.28** |  |  |
| Item 18 | 0.40 | 0.66 |  |  |
| **Item 19** |  |  | **0.10** | **0.32** |
| **Item 20** | **0.02** | **0.16** |  |  |
| **Item 21** | **0.12** | **0.35** |  |  |
| **Item 22** | **0.12** | **0.31** |  |  |
| **Item 23** |  |  | **0.11** | **0.33** |
| Item 24 |  |  | 0.35 | 0.43 |
| **Item 25** | **0.01** | **0.19** |  |  |
| *Note*: Bolded items removed due to low communalities | | | | |
